# The Effect of Nurse Aide Retention on Ohio's Nursing Home Resident Care Experience Scores: A Facility-Level Analysis

**DOI:** 10.1093/geroni/igab046.354

**Published:** 2021-12-17

**Authors:** Katherine Kennedy

**Affiliations:** LTSS COIN Providence VA Medical Center

## Abstract

The objective of the study was to analyze whether higher nurse aide retention was related to better resident care experiences using an overall score and seven domain scores among a sample of Ohio nursing homes. The 2017 Ohio Biennial Survey of Long-Term Care Facilities was used in combination with the 2017 Ohio Nursing Home Resident Satisfaction Survey. These data were merged with the Ohio Medicaid Cost Report, Certification and Survey Provider Enhanced Reports, LTC Focus, Area Health Resource File, Rural Urban Commuting Area data, and Payroll-based Journal Public Use Files. The analytic sample (N=690) represents freestanding facilities with a full-year cost report. The analysis included means and frequencies, ANOVAs with Tukey adjustments, and linear regressions that controlled for heteroskedasticity. Quartiles of the CNA retention rate were used to define four groups: low, medium, high, and extremely high. After controlling for facility and county characteristics, facilities with high CNA retention (61-72%) had significantly higher overall resident care experience scores by 1.27 percentage points and better environment scores by 1.35 percentage points compared to those with low CNA retention (0-48%). Medium retention (49-60%) also had significantly better environment scores than low retention. Compared to the high retention group, facilities with extremely high retention (73%+) had significantly lower scores for the overall resident care experience, facility culture, caregivers, and spending time. Maintaining a high retention rate of CNAs is important, but there were surprising negative effects from having extremely high retention potentially due to high burnout or poor person-job fit.

